# NRF2 Deficiency Disrupts Mitochondrial Homeostasis via NDUFS7 in Trabecular Meshwork

**DOI:** 10.34133/research.1203

**Published:** 2026-03-20

**Authors:** Xuejing Yan, Shen Wu, Xiaowei Fan, Qian Li, Yufei Teng, Ningli Wang, Jingxue Zhang

**Affiliations:** ^1^ Beijing Institute of Ophthalmology, Beijing Tongren Eye Center, Beijing Tongren Hospital, Beijing Key Laboratory of Intelligent Diagnosis Technology and Equipment for Optic Nerve-Related Eye Diseases, Capital Medical University, Beijing 100730, China.; ^2^ National Engineering Research Center for Ophthalmology, Beijing 100730, China.; ^3^ Henan Academy of Innovations in Medical Science, Zhengzhou 450000, China.

## Abstract

The trabecular meshwork (TM) plays a pivotal role in maintaining intraocular pressure (IOP) by regulating aqueous humor outflow. Nuclear factor erythroid 2-related factor 2 (NRF2) was identified as a key transcriptional controller of TM redox balance and mitochondrial function. Transcriptomic profiling of tert-butyl hydroperoxide (tBHP)-induced oxidative injury revealed NRF2 pathway involvement in TM cellular defense. NRF2 knockout (KO) mice exhibited impaired aqueous humor dynamics, elevated IOP, and TM oxidative damage. In vitro, NRF2 knockdown aggravated oxidative stress and mitochondrial dysfunction, whereas NRF2 overexpression mitigated tBHP-induced cytotoxicity. The results of the gene set enrichment analysis (GSEA) indicated enrichment of oxidative phosphorylation pathway in NRF2-deficient cells. Chromatin immunoprecipitation sequencing (ChIP-seq) confirmed NDUFS7 as a direct NRF2 target essential for mitochondrial complex I integrity. Restoration of NDUFS7 expression in NRF2-deficient TM cells or KO mice rescued mitochondrial impairment. Collectively, these findings establish the NRF2/NDUFS7 axis as a central defense mechanism protecting TM from oxidative injury and suggest potential therapeutic strategies for glaucoma-associated ocular hypertension.

## Introduction

Glaucoma, as a heterogeneous group of optic neuropathies, constitutes a pathological process marked by progressive retinal ganglion cell (RGC) degeneration, retinal nerve fiber layer thinning, and, ultimately, irreversible visual field loss [1]. Epidemiological projections estimate that over 100 million people worldwide will be affected within the next 2 decades [2]. The principal pathogenic driver of glaucoma is elevated intraocular pressure (IOP), primarily regulated by the trabecular meshwork (TM), a specialized tissue that controls aqueous humor outflow through complex cellular signaling and extracellular matrix remodeling [3]. Current therapeutic strategies largely focus on lowering IOP through pharmacologic or surgical interventions that either enhance aqueous humor drainage or suppress its production. However, these approaches do not address the underlying molecular pathology, halt disease progression, or restore lost vision [4]. Thus, elucidating the mechanisms underlying TM cell injury and developing long-term strategies to preserve TM function are of critical importance.

An imbalance between reactive oxygen species (ROS) generation and antioxidant capacity drives oxidative stress, which in turn markedly raises IOP in glaucoma. Mitochondria, as both the primary source and target of ROS, play a central role in this pathological cascade. Impaired oxidative phosphorylation (OXPHOS) amplifies ROS generation, establishing a vicious cycle that promotes apoptosis and compromises aqueous humor outflow. TM cells from glaucoma patients exhibit diminished mitochondrial membrane potential, swollen and structurally deformed mitochondria, and disrupted inner membrane cristae [5]. Moreover, mitochondrial gene mutations have been linked to increased susceptibility to IOP elevation and glaucoma [6,7]. In glucocorticoid-induced models of TM injury, mitochondria display fragmentation, excessive ROS accumulation, and impaired adenosine triphosphate (ATP) synthesis [8]. Similarly, our previous studies demonstrated mitochondrial dysfunction in mechanically stretched TM cells, mimicking glaucomatous stress [9]. Collectively, these findings identify mitochondrial impairment as a pivotal driver of TM pathology in glaucoma. Nevertheless, the precise mechanisms underlying mitochondrial damage in TM cells and how this dysfunction translates to impaired outflow regulation remain poorly understood.

As a pivotal transcription factor, nuclear factor erythroid 2-related factor 2 (NRF2) governs the central pathways responsible for maintaining cellular redox homeostasis and mitochondrial integrity. NRF2 deficiency induces mitochondrial depolarization, ATP depletion, and impaired respiration, whereas its activation enhances mitochondrial performance [10]. Mechanistically, NRF2 modulates mitochondrial function by regulating genes involved in energy metabolism and respiration [11,12]. Although these findings provide valuable context and highlight the therapeutic potential of NRF2 activation in diverse disease models involving mitochondrial impairment, the exact contribution of NRF2 in TM cells and its role in mitochondrial dysfunction in glaucoma remain unexplored.

Our chromatin immunoprecipitation sequencing (ChIP-seq) analysis following NRF2 overexpression revealed NDUFS7 as a direct downstream target of NRF2. NDUFS7 is essential for maintaining mitochondrial complex I (CI) structure and enzymatic activity (NADH: ubiquinone oxidoreductase), serving as a key component of the iron–sulfur (Fe–S) cluster-binding module that facilitates electron transfer within the electron transport chain (ETC) [13]. Mutations in NDUFS7 disrupt CI assembly and are associated with severe mitochondrial diseases such as Leigh syndrome [14]. These findings implicate NDUFS7 as a potential mediator of NRF2-driven mitochondrial homeostasis in TM cells, suggesting new therapeutic opportunities for mitochondrial dysfunction-associated glaucomatous degeneration.

Here, we uncover a previously unrecognized route through which NRF2 protects TM cells from tert-butyl hydroperoxide (tBHP)-induced oxidative injury through direct regulation of mitochondrial function. Transcriptomic analysis revealed marked alterations in the OXPHOS pathway in NRF2 knockout (KO) TM cells compared with wild-type (WT) controls. ChIP-seq confirmed direct binding of NRF2 to the NDUFS7 promoter, indicating a previously unrecognized role of NRF2 in sustaining mitochondrial bioenergetics. Together, these findings redefine NRF2 as a dual regulator of cellular defense governing both antioxidant responses and mitochondrial function in TM cells. The elucidation of the NRF2/NDUFS7 axis offers promising therapeutic potential for glaucoma by concurrently mitigating oxidative stress and restoring mitochondrial integrity.

## Results

### NRF2 signaling participates in oxidative injury process and its deficiency causes TM injury

**Fig. 1.**
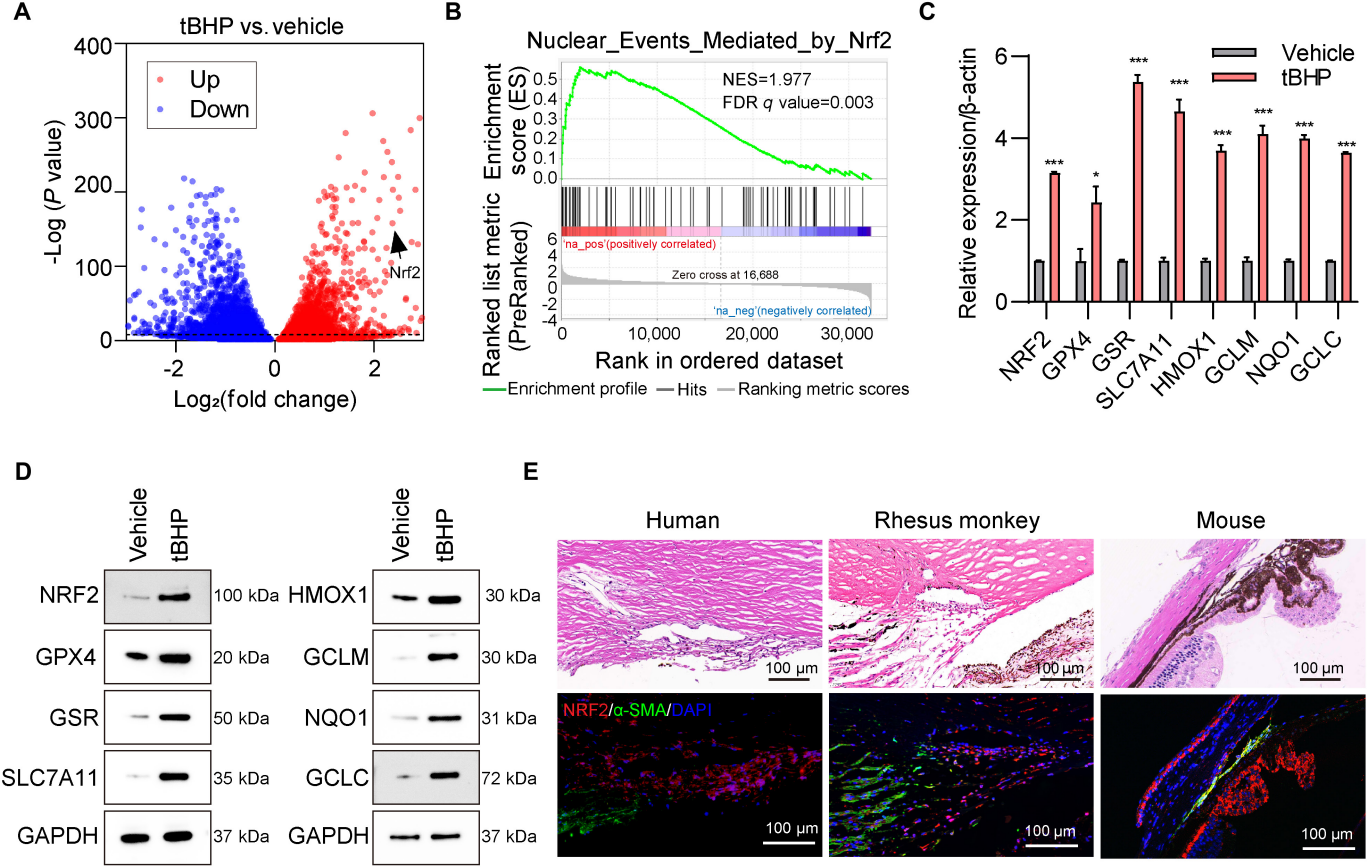
NRF2 activation in response to oxidative stress in the trabecular meshwork (TM). (A) Volcano plot of differentially expressed genes (DEGs) in human TM cells with or without tert-butyl hydroperoxide (tBHP) treatment. (B) Gene set enrichment analysis (GSEA) of human TM cells with or without tBHP treatment. (C) mRNA expression of NRF2, GPX4, GSR, SLC7A11, HMOX1, GCLM, NQO1, and GCLC in human TM cells with or without tBHP exposure. *P < 0.05, ***P < 0.001. (D) Protein expression of NRF2, GPX4, GSR, SLC7A11, HMOX1, GCLM, NQO1, and GCLC in human TM cells with or without tBHP exposure. (E) Immunofluorescence staining of NRF2 (red) and the TM marker α-SMA (green) in human corneoscleral rings, rhesus monkey eyes, and mouse eyes. Nuclei are counterstained with 4′,6-diamidino-2-phenylindole (DAPI) (blue).

In our previous transcriptomic analysis of tBHP-treated TM cells, we identified numerous differentially expressed genes (DEGs) (Fig. 1A). Given our focus on oxidative stress-related mechanisms, we performed gene set enrichment analysis (GSEA) on these DEGs. This revealed marked enrichment in several pathways broadly associated with oxidative injury, including hypoxia, ferroptosis, and oxidative stress response (Fig. S1A to C). To more precisely delineate the regulatory basis of cellular antioxidant defense, we narrowed our analysis to pathways directly involved in the transcriptional control of antioxidant responses. This approach highlighted several NRF2 centric pathways, including NRF2 regulation of antioxidant detoxification enzymes, KEAP1–NFE2L2 pathway, NRF2–ARE regulation, NRF2 pathway, and Nuclear_Events_Mediated_by_Nrf2 (Fig. 1B and Fig. S2A to D). In line with this hypothesis, both mRNA (Fig. 1C) and protein (Fig. 1D) levels of NRF2 and its downstream antioxidant target genes were elevated following tBHP treatment. Moreover, strong NRF2 expression was consistently detected in the TM of human, rhesus monkey, and mouse ocular tissues (Fig. 1E). Collectively, our findings indicate that NRF2 plays a pivotal role in modulating TM cellular homeostasis and may contribute to TM pathology.

To elucidate the in vivo functional consequences of NRF2 deficiency, we generated NRF2 KO mice (Fig. S3A). Gadolinium-enhanced MRI revealed impaired aqueous humor circulation in KO mice, as evidenced by delayed clearance of contrast from the anterior chamber relative to WT controls (Fig. S3B). Quantitative assessment confirmed a marked reduction in aqueous outflow facility in KO mice (Fig. S3C), accompanied by a pronounced increase in IOP (Fig. S3D). Immunofluorescence costaining for alpha-smooth muscle actin (α-SMA) and malondialdehyde (MDA), a lipid peroxidation marker, showed enhanced MDA accumulation within the TM of KO mice (Fig. S3E). Furthermore, terminal deoxynucleotidyl transferase–mediated deoxyuridine triphosphate nick end labeling (TUNEL) assays combined with α-SMA labeling revealed an increased number of apoptotic cells within the anterior angle (Fig. S3F).

Together, these data suggest that loss of NRF2 disrupts TM redox homeostasis, triggering oxidative damage, TM cell apoptosis, and impaired aqueous humor drainage, which culminate in elevated IOP.

### NRF2 protects TM cells from oxidative stress injury

**Fig. 2.**
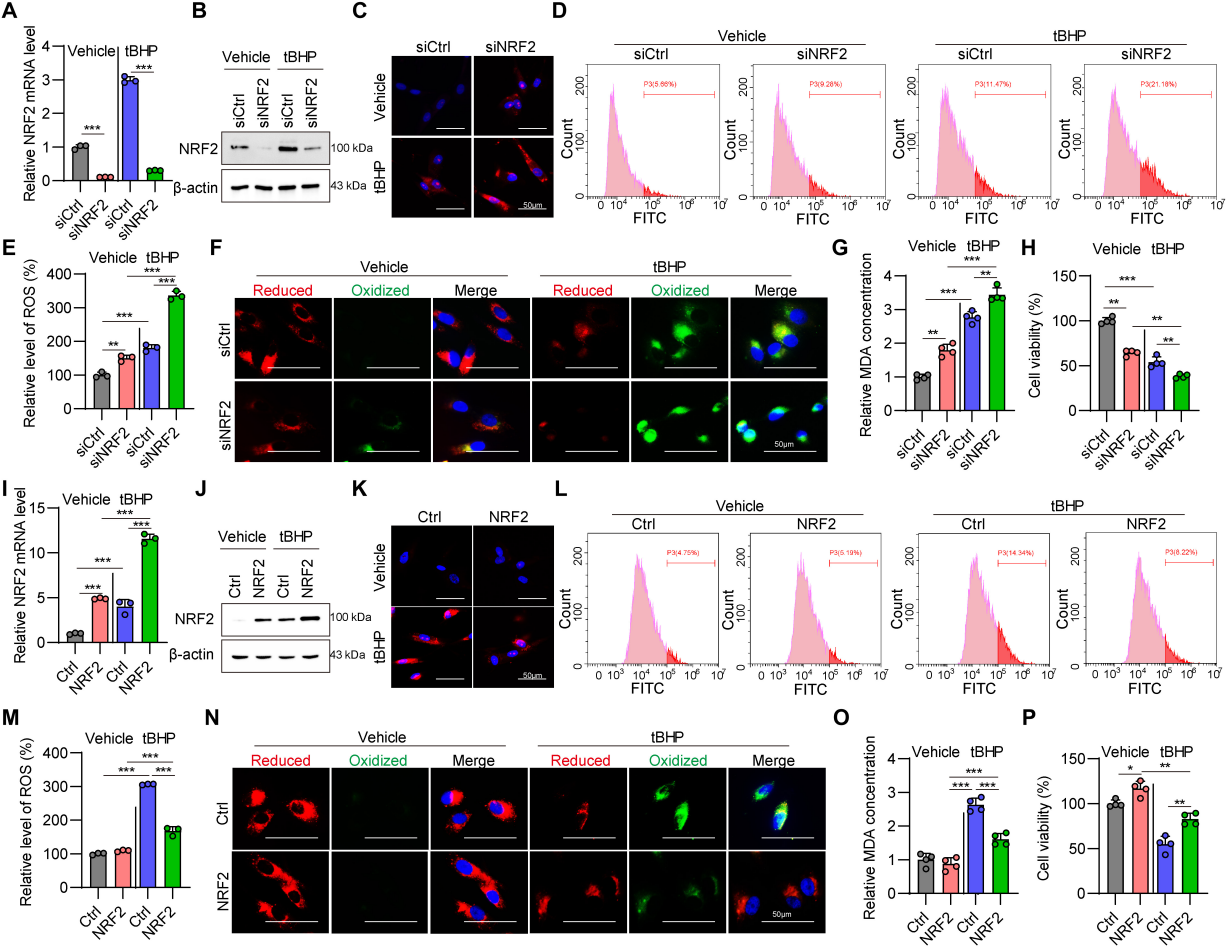
NRF2 protects TM cells from tBHP-induced oxidative stress damage. (A) Relative NRF2 mRNA expression in TM cells under NRF2 knockdown with or without tBHP exposure detected by quantitative PCR (qPCR). ***P < 0.001. (B) NRF2 protein expression in TM cells under NRF2 knockdown with or without tBHP exposure tested using Western blot. (C) Dihydroethidium (DHE) staining in TM cells under NRF2 knockdown with or without tBHP exposure. (D) Measurement of reactive oxygen species (ROS) levels by flow cytometry in TM cells under NRF2 knockdown with or without tBHP exposure. (E) Quantification of the data shown in (D). **P < 0.01, ***P < 0.001. (F) BODIPY 581/591 staining of TM cells under NRF2 knockdown with or without tBHP exposure. (G) Measurement of malondialdehyde (MDA) concentration in TM cells under NRF2 knockdown with or without tBHP exposure. **P < 0.01, ***P < 0.001. (H) Cell viability detection by CCK-8 in TM cells under NRF2 knockdown with or without tBHP exposure. **P < 0.01, ***P < 0.001. (I) Relative NRF2 mRNA expression in TM cells under NRF2 overexpression with or without tBHP exposure detected by qPCR. ***P < 0.001. (J) Western blot results of NRF2 protein expression in TM cells under NRF2 overexpression with or without tBHP exposure. (K) DHE staining in TM cells under NRF2 overexpression with or without tBHP exposure. (L) Measurement of ROS levels by flow cytometry in TM cells under NRF2 overexpression with or without tBHP exposure. (M) Quantification of the data shown in (L). ***P < 0.001. (N) BODIPY 581/591 staining in TM cells under NRF2 overexpression with or without tBHP exposure. (O) MDA concentration in TM cells under NRF2 overexpression with or without tBHP exposure. ***P < 0.001. (P) Cell viability in TM cells under NRF2 knockdown with or without tBHP exposure. *P < 0.05, **P < 0.01.

To validate the functional role of NRF2 in TM cells, we performed small interfering RNA (siRNA)-mediated NRF2 knockdown with or without tBHP exposure. Quantitative polymerase chain reaction (qPCR) and Western blot analysis confirmed marked decrease of NRF2 mRNA (Fig. 2A) and protein expression (Fig. 2B) compared with scrambled controls. NRF2-deficient TM cells exhibited increased superoxide accumulation detected by dihydroethidium (DHE) staining (Fig. 2C), elevated ROS generation (Fig. 2D and E), and redox imbalance visualized by BODIPY staining (Fig. 2F). Moreover, lipid peroxidation, indicated by MDA content, was markedly higher in NRF2-silenced cells (Fig. 2G). To assess the role of NRF2 in maintaining glutathione redox balance, intracellular GSH (reduced form of glutathione) levels and the GSH/GSSG (oxidized glutathione) ratio were examined in TM cells under basal conditions and following oxidative stress. NRF2 knockdown resulted in a marked reduction in intracellular GSH levels, which was evident under basal conditions and became more pronounced upon tBHP treatment (Fig. S4A). Consistently, depletion of NRF2 markedly decreased the GSH/GSSG ratio in both untreated and tBHP-exposed cells, indicating a shift toward a more oxidized intracellular redox state (Fig. S4B). This oxidative stress response led to a notable decline in TM cell viability (Fig. 2H). These results establish that NRF2 deficiency induces oxidative stress mediated cytotoxicity in TM cells.

To assess whether NRF2 activation could mitigate oxidative injury, primary human TM cells were treated with tBHP in the presence or absence of NRF2 overexpression. NRF2 up-regulation was verified at both transcriptional (Fig. 2I) and protein levels (Fig. 2J). In tBHP-treated cells, NRF2 overexpression markedly attenuated superoxide and ROS accumulation (Fig. 2K to M) and restored redox equilibrium (Fig. 2N) and cellular glutathione homeostasis (Fig. S4C and D), while reducing lipid peroxidation (Fig. 2O). Importantly, these antioxidant effects translated into preserved TM cell viability, with NRF2 overexpression effectively reversing tBHP-induced cytotoxicity (Fig. 2P).

Altogether, these findings demonstrate that NRF2 is sufficient to confer cytoprotection against oxidative stress in TM cells.

### NRF2 deficiency disrupts mitochondrial function in TM cells

**Fig. 3.**
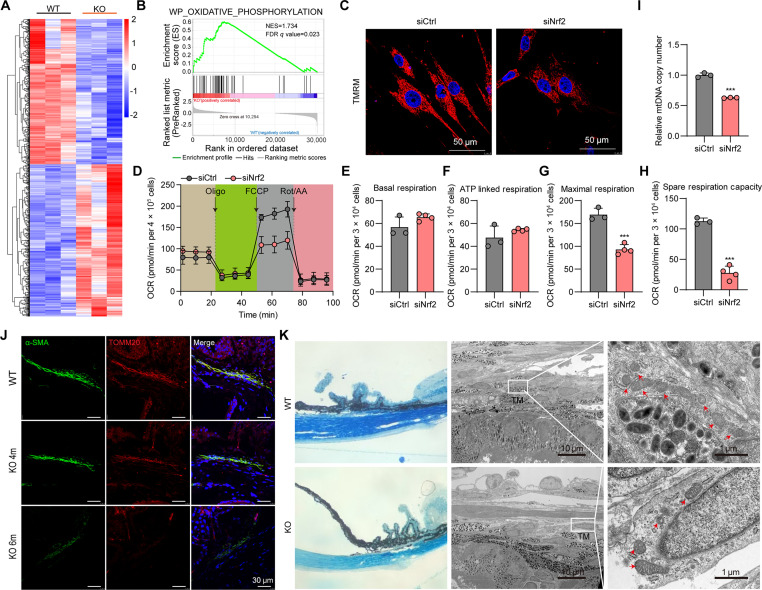
Lack of NRF2 leads to mitochondrial dysfunction. (A) Heatmap of DEGs in wild-type (WT) and NRF2 knockout (KO) mice. (B) GSEA between the NRF2 KO and WT groups. (C) TMRE staining in TM cells with or without NRF2 knockdown. (D) Extracellular flux analysis (seahorse) shows a representative trace of the oxygen consumption rate (OCR) following the mitochondrial stress test protocol in the siCtrl and siNRF2 groups. (E to H) Histogram profiles of the calculated basal respiration (E), adenosine triphosphate (ATP)-linked respiration (F), maximal respiration (G), and spare respiration capacity (H). ***P < 0.001. (I) Measurement of mitochondrial DNA (mtDNA) copy number in TM cells with or without NRF2 knockdown. ***P < 0.001. (J) Immunofluorescence staining of the TM marker α-SMA (green) and TOMM20 (red) in the anterior chamber angle tissue in WT, NRF2 KO-4 month, and NRF2 KO-6-month mice. (K) Transmission electron microscopy (TEM) analysis of the mitochondrial structure in TM cells in WT and NRF2 KO mice.

To elucidate the mechanisms by which NRF2 deficiency mediates injury in TM cells, we conducted RNA sequencing (RNA-seq) on anterior chamber angle tissues from NRF2 KO mice. A pronounced transcriptomic reprogramming was observed in these tissues, as illustrated in the heatmap presented in Fig. 3A. GSEA further revealed marked enrichment of pathways related to OXPHOS (Fig. 3B), indicating that NRF2 deficiency disrupts cellular energy metabolism. Mitochondrial membrane potential analysis using tetramethylrhodamine ethyl ester perchlorate (TMRE) staining (Fig. 3C) demonstrated a pronounced reduction in membrane potential upon NRF2 knockdown. Consistently, Seahorse metabolic flux assays (Fig. 3D) confirmed impaired mitochondrial function. Although basal respiration (Fig. 3E) and ATP-linked respiration (Fig. 3F) were unaffected, NRF2 knockdown markedly reduced maximal respiratory capacity (Fig. 3G) and spare respiratory capacity (Fig. 3H), signifying diminished mitochondrial resilience to stress. Further, NRF2 silencing markedly decreased mitochondrial DNA content (Fig. 3I), indicating compromised mitochondrial biogenesis. Immunofluorescence staining of the mitochondrial marker TOMM20 in NRF2 KO mice revealed comparable expression to WT controls at 4 months but a substantial decline by 6 months (Fig. 3J), suggesting an age-dependent loss of mitochondrial mass in the absence of NRF2. Transmission electron microscopy (TEM) of TM tissue from NRF2 KO mice (Fig. 3K) corroborated these findings, showing ultrastructural mitochondrial abnormalities indicative of dysfunction.

Collectively, these results demonstrate that NRF2 deficiency disrupts mitochondrial energetics, structural integrity, and adaptive capacity, ultimately impairing the mitochondrial health and stress response of TM cells.

### NRF2 binds to the promoter of NDUFS7 to regulate its expression

**Fig. 4.**
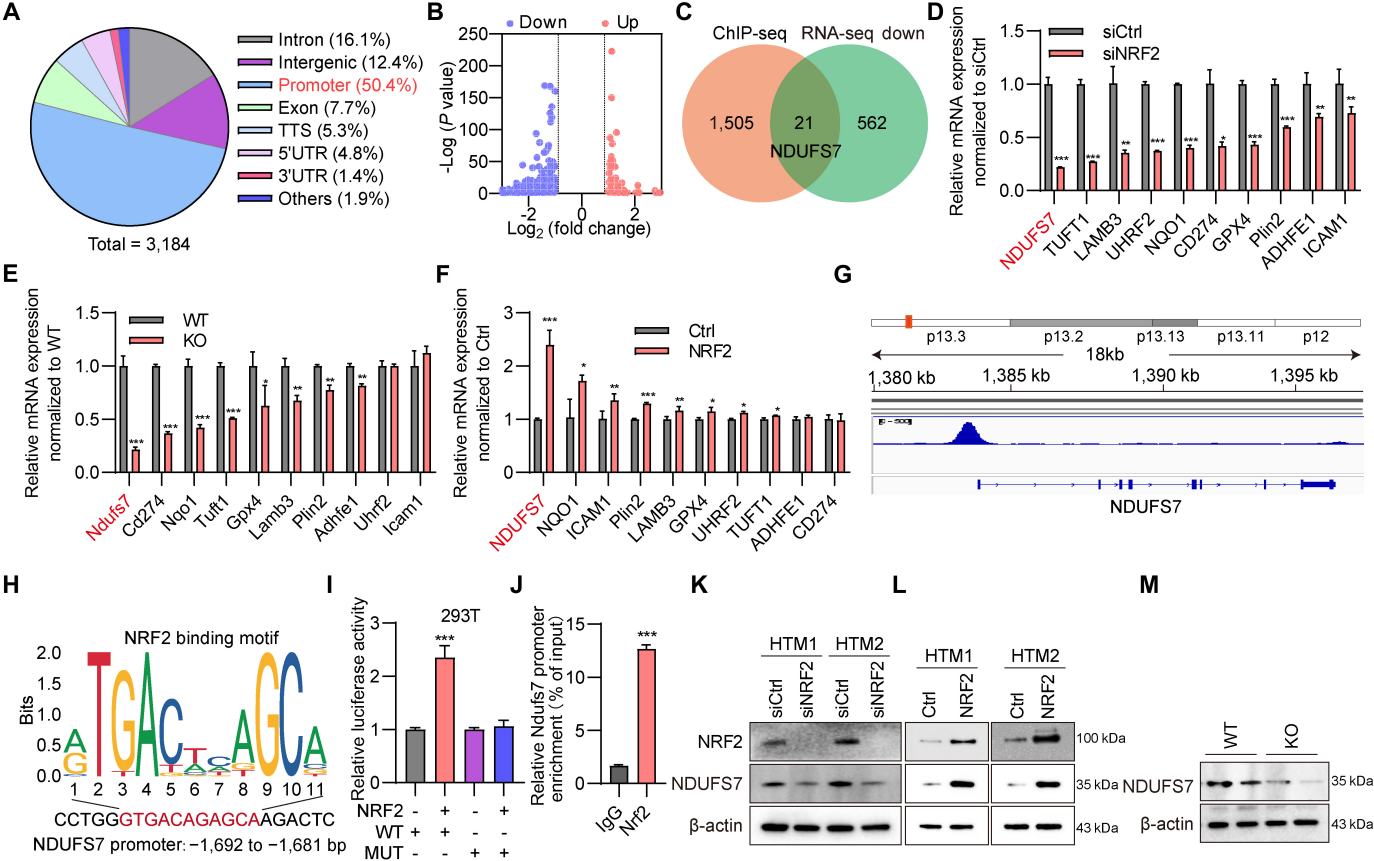
NRF2 regulates NDUFS7 expression by directly binding to its promoter region. (A) Pie chart of the results of chromatin immunoprecipitation sequencing (ChIP-seq) analysis showing the genomic distribution of NRF2 binding peaks. (B) Volcano plot displaying differentially expressed genes following NDUFS7 knockdown. (C) Venn diagram illustrating the overlap between genes with direct NRF2 binding peaks and those down-regulated upon NRF2 knockdown in RNA sequencing (RNA-seq) data. (D) mRNA expression levels of 10 candidate target genes in TM cells after NRF2 knockdown detected by quantitative real-time PCR (qRT-PCR). *P < 0.05, **P < 0.01, ***P < 0.001. (E) mRNA expression levels of the 10 candidate genes in the anterior chamber angle tissue of NRF2 KO mice and WT littermates detected by qRT-PCR. *P < 0.05, **P < 0.01, ***P < 0.001. (F) mRNA expression levels of the 10 candidate genes in TM cells following NRF2 overexpression. *P < 0.05, **P < 0.01, ***P < 0.001. (G) Representative ChIP-seq track showing a specific NRF2 binding peak at the promoter region of the NDUFS7 gene. (H) NRF2 binding motif (up) and its specific location within the NDUFS7 promoter sequence (down). (I) Relative luciferase activity in different groups detected by luciferase reporter assay. Constructs containing either the WT or mutant (Mut) NDUFS7 promoter were cotransfected with an NRF2 expression vector or an empty vector. ***P < 0.001. (J) ChIP-qPCR analysis of *NRF2* enrichment at the *NDUFS7* promoter. Immunoglobulin G (IgG) served as the negative control. Enrichment efficiency is presented as the percentage of target DNA fragments relative to the input. ***P < 0.001. (K) Protein levels of NRF2 and NDUFS7 in 2 independent strains of human TM cells following NRF2 knockdown. (L) Protein levels of NRF2 and NDUFS7 in 2 independent strains of human TM cells following NRF2 overexpression. (M) Protein levels of NDUFS7 in the anterior chamber angle tissue of NRF2 KO mice versus WT controls.

To define the mechanistic basis of NRF2 regulation in TM cells, we conducted ChIP-seq in NRF2-overexpressing TM cells to recognize genome-wide NRF2 binding sites. NRF2 binding was broadly distributed, with the promoter region showing the highest occupancy (50.4%) (Fig. 4A). Integration of the ChIP-seq data with RNA-seq results from NRF2 knockdown TM cells (Fig. 4B) identified 129 up-regulated and 583 down-regulated genes following NRF2 depletion. Cross-analysis revealed 21 overlapping genes that were both bound by NRF2 and down-regulated upon its knockdown (Fig. 4C), representing potential direct NRF2 targets. From these candidates, 10 genes containing canonical NRF2 binding motifs were selected for validation. Among them, NDUFS7 exhibited the most marked transcriptional reduction following NRF2 knockdown (Fig. 4D) and in NRF2 KO mice (Fig. 4E), and conversely, strong up-regulation upon NRF2 overexpression (Fig. 4F). ChIP-seq data revealed a clear NRF2 binding peak at the NDUFS7 locus (Fig. 4G), with motif analysis pinpointing NRF2 interaction at the –1,692 to –1,681 bp region upstream of the NDUFS7 transcription start site (Fig. 4H), indicating direct promoter binding. Functional validation using a dual-luciferase reporter assay confirmed that NRF2 activates the NDUFS7 promoter, while mutation of its binding site markedly attenuated promoter activity (Fig. 4I). ChIP-qPCR further verified NRF2 occupancy on the NDUFS7 promoter (Fig. 4J). In 2 independent TM cell strains, NRF2 knockdown markedly reduced NDUFS7 protein expression (Fig. 4K), whereas NRF2 overexpression enhanced it (Fig. 4L). Consistent with in vitro findings, NRF2 KO mice showed a substantial reduction in NDUFS7 protein levels in the anterior angle tissue (Fig. 4M).

Taken together, these data establish that NRF2 directly binds to the NDUFS7 promoter and transcriptionally regulates the NDUFS7 expression, thereby sustaining mitochondrial gene expression and function in TM cells both in vitro and in vivo.

### Lack of NDUFS7 induces mitochondrial dysfunction

**Fig. 5.**
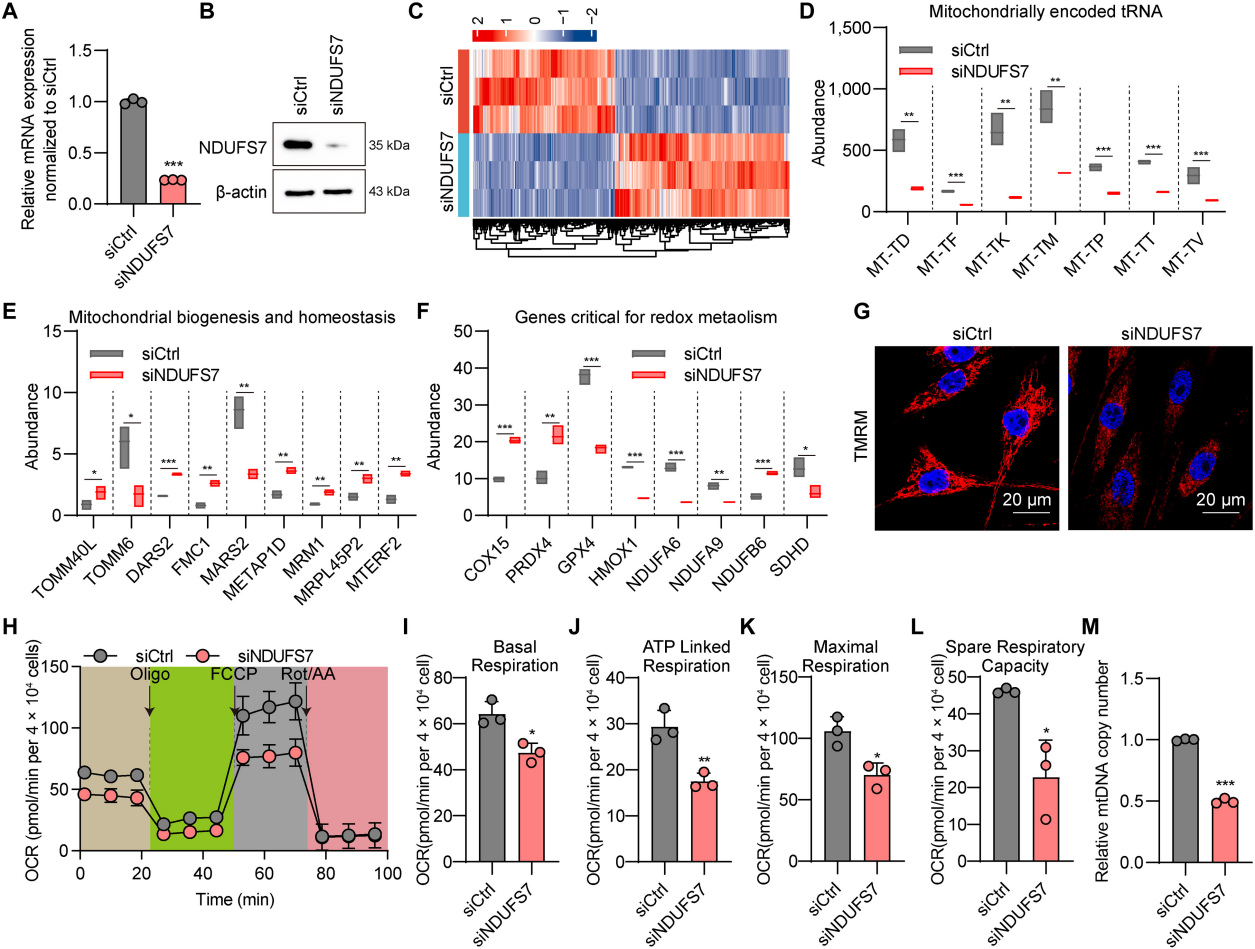
Lack of NDUFS7 results in mitochondrial dysfunction. (A) Relative NDUFS7 mRNA expression in TM cells under NDUFS7 knockdown. ***P < 0.001. (B) NDUFS7 protein expression in TM cells under NDUFS7 knockdown. (C) Heatmap of DEGs in the siCtrl and siNDUFS7 groups. (D) RNA abundance of genes related in mitochondrially encoded tRNAs. **P < 0.01, ***P < 0.001. (E) RNA abundance of genes related in mitochondrial biogenesis and homeostasis. *P < 0.05, **P < 0.01, ***P < 0.001. (F) RNA abundance of genes critical for redox metabolism. *P < 0.05, **P < 0.01, ***P < 0.001. (G) TMRE staining in TM cells following NDUFS7 knockdown. (H) Seahorse assay showing a representative trace of the OCR following the mitochondrial stress test protocol in the siCtrl and siNDUFS7 groups. (I to L) Histogram profiles of calculated basal respiration (I), ATP-linked respiration (J), maximal respiration (K), and spare respiration capacity (L). *P < 0.05, **P < 0.01. (M) Measurement of mtDNA copy number in TM cells following NDUFS7 knockdown. ***P < 0.001.

qPCR analysis revealed a marked reduction in NDUFS7 mRNA expression following gene knockdown, confirming efficient silencing (Fig. 5A). Consistently, Western blot analysis demonstrated a decrease in NDUFS7 protein levels in knockdown TM cells (Fig. 5B), validating down-regulation at both mRNA and protein levels. Transcriptomic profiling of NDUFS7-deficient TM cells revealed extensive gene expression alterations, as illustrated by the heatmap in Fig. 5C. Specifically, marked dysregulation was observed in genes encoding mitochondrially derived tRNAs (Fig. 5D), a broad range of genes involved in mitochondrial biogenesis and homeostasis (Fig. 5E), and those governing redox metabolism, including core subunits of the OXPHOS system (NDUFA6, NDUFA9, SDHD, and COX15) and major antioxidant mediators (PRDX4, GPX4, and HMOX1) (Fig. 5F). This coordinated transcriptional pattern underscores a state of severe mitochondrial impairment following NDUFS7 depletion. Mitochondrial function was next assessed using TMRE staining, which showed a pronounced decline in mitochondrial membrane potential in NDUFS7-knockdown TM cells, indicating functional compromise (Fig. 5G). Seahorse assay analysis further confirmed mitochondrial dysfunction, as reflected by altered oxygen consumption rates (OCRs) (Fig. 5H). Specifically, basal respiration was markedly reduced (Fig. 5I), accompanied by diminished ATP-linked respiration (Fig. 5J), reflecting impaired cellular energy production. Moreover, maximal respiratory capacity was markedly decreased (Fig. 5K), indicating limited ability to meet energy demands under stress, while spare respiratory capacity was also reduced (Fig. 5L), highlighting the loss of mitochondrial adaptability and resilience. Additionally, mitochondrial DNA content was substantially decreased in the NDUFS7-knockdown group (Fig. 5M).

Collectively, these findings demonstrate that NDUFS7 is indispensable for preserving mitochondrial integrity, bioenergetic efficiency, and adaptive capacity in TM cells.

### NRF2 deficiency induced mitochondrial dysfunction dependent on NDUFS7

**Fig. 6.**
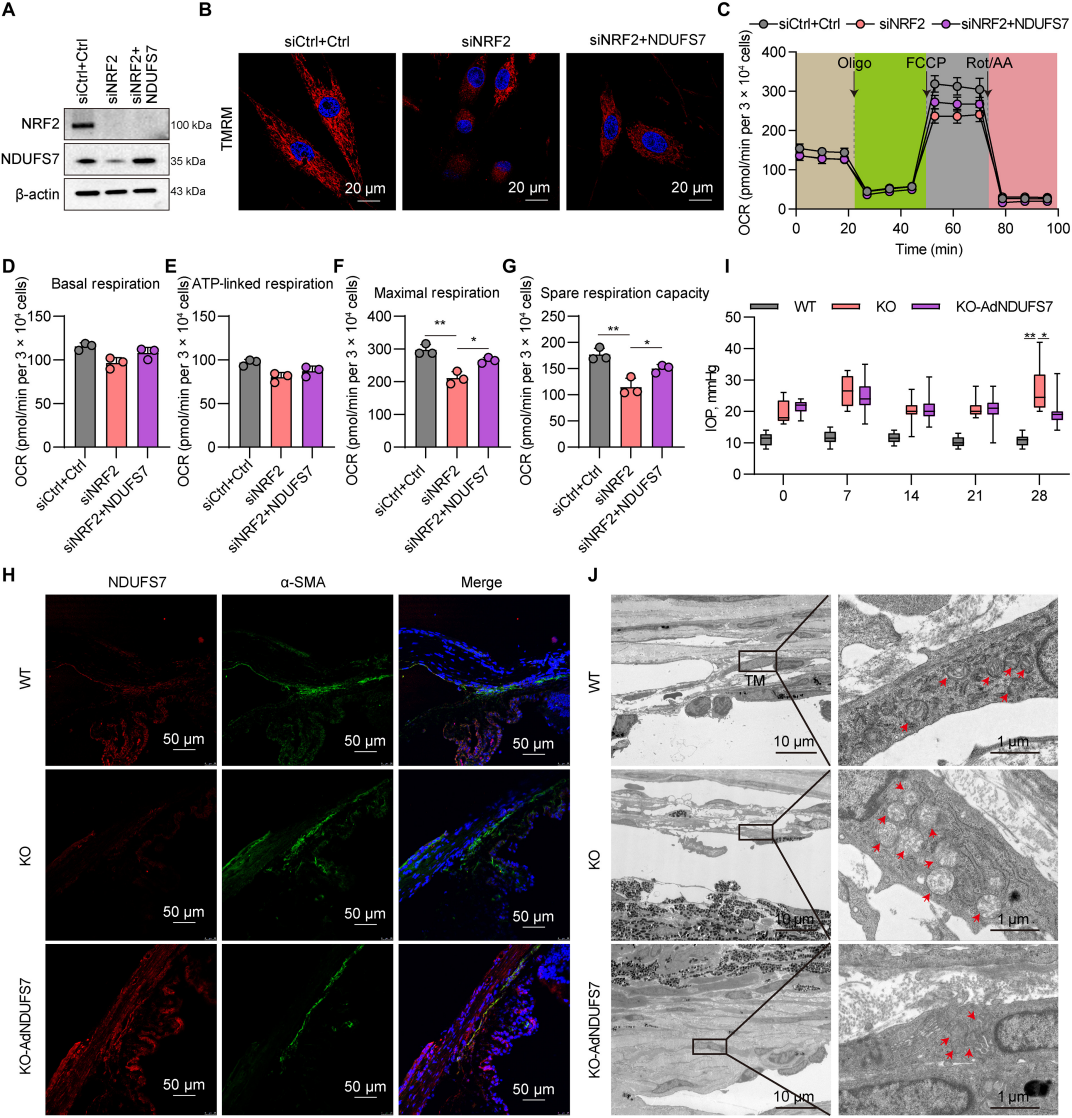
Supplementation of NDUFS7 alleviates mitochondrial dysfunction caused by NRF2 deficiency. (A) Protein levels of NRF2 and NDUFS7 in TM cells in siCtrl+Ctrl, siNRF2, and siNRF2+NDUFS7 groups. (B) TMRE staining in TM cells in the siCtrl+Ctrl, siNRF2, and siNRF2+NDUFS7 groups. (C) Seahorse assay shows a representative trace of the OCR following the mitochondrial stress test protocol in siCtrl+Ctrl, siNRF2, and siNRF2+NDUFS7 groups. (D to G) Histogram profiles of the calculated basal respiration (D), ATP-linked respiration (E), maximal respiration (F), and spare respiration capacity (G). *P < 0.05, **P < 0.01. (H) Immunofluorescence staining of the TM markers α-SMA (green) and NDUFS7 (red) in the anterior chamber angle tissue in the WT, KO, and KO-AdNDUFS7 groups. (I) Intraocular pressure (IOP) measurement in WT, NRF2 KO mice (KO), and NRF2 KO mice receiving an intracamerally injection of NDUFS7 adenovirus (KO-AdNDUFS7). *P < 0.05, **P < 0.01. (J) TEM analysis of mitochondria structure in TM tissue in the WT, KO, and KO-AdNDUFS7 groups.

To investigate whether NDUFS7 supplementation can counteract NRF2 knockdown-induced mitochondrial dysfunction in TM cells, we overexpressed NDUFS7 in NRF2 knockdown TM cells. Western blot analysis confirmed that NRF2 protein levels were markedly reduced in the NRF2 knockdown group relative to controls (Fig. 6A). Importantly, NRF2 expression remained markedly low even after NDUFS7 supplementation, indicating that NDUFS7 does not reverse NRF2 silencing. Similarly, NDUFS7 protein levels were markedly decreased in the NRF2 knockdown group (Fig. 6A). However, NDUFS7 supplementation in NRF2-deficient cells partially restored NDUFS7 protein expression compared with controls (Fig. 6A). As shown in Fig. 6B, the mitochondrial membrane potential was markedly diminished in NRF2 knockdown cells. NDUFS7 supplementation markedly alleviated this decline, suggesting a partial rescue of mitochondrial function. To further assess mitochondrial performance, Seahorse assays were conducted across all groups (Fig. 6C). NRF2 knockdown cells exhibited pronounced mitochondrial dysfunction, characterized by reduced maximal respiratory capacity and spare respiratory capacity (Fig. 6F and G), while basal respiration and ATP-linked respiration remained unchanged (Fig. 6D and E), consistent with prior observations (Fig. 3E and F). Notably, NDUFS7 supplementation markedly ameliorated these deficits, partially restoring both maximal and spare respiratory capacities (Fig. 6F and G). Collectively, these findings indicate that NDUFS7 supplementation partially rescues NRF2 deficiency-induced mitochondrial dysfunction.

We next evaluated whether NDUFS7 supplementation could mitigate mitochondrial damage associated with NRF2 deficiency in vivo using NRF2 KO mice. Adenoviral-mediated delivery of NDUFS7 was confirmed by a robust increase in NDUFS7 immunofluorescence intensity at the anterior chamber angle of NRF2 KO mice compared to untreated KO and WT controls (Fig. 6H). This molecular restoration was accompanied by functional improvement. IOP, monitored on days 0, 7, 14, 21, and 28 postinjection, showed that the elevated IOP observed in NRF2 KO mice was markedly reduced by day 28 following NDUFS7 treatment (Fig. 6I). Consistently, TEM provided ultrastructural confirmation of mitochondrial recovery, revealing improved mitochondrial morphology in TM of NDUFS7-treated KO mice (Fig. 6J).

Together, these data demonstrate that restoring NDUFS7 expression effectively rescues mitochondrial defects and ameliorates the pathological IOP elevation associated with NRF2 loss.

## Discussion

This study delineates a previously unrecognized NRF2/NDUFS7 regulatory axis that is essential for maintaining mitochondrial homeostasis in TM cells. The findings provide mechanistic insight into how oxidative stress drives TM dysfunction and elevates IOP in glaucoma. Specifically, NRF2 deficiency was shown to impair mitochondrial function through down-regulation of NDUFS7, a core subunit of mitochondrial CI, leading to oxidative damage, disrupted aqueous humor outflow, and TM cell apoptosis. Restoration of NDUFS7 expression effectively rescued mitochondrial defects in NRF2-deficient TM cells, underscoring its pivotal role in NRF2-mediated cyto-protection.

Redox homeostasis is a fundamental regulator of diverse cellular processes, including metabolism, immune response, apoptosis, proliferation, and differentiation [15]. Previous work from our group identified oxidative stress as a major contributor to the pathogenesis of primary open-angle glaucoma (POAG), with DEGs in POAG patients being markedly enriched in oxidative stress-related pathways [16]. Consistent with these findings, our recently published work demonstrated that bioinformatic analysis of TM tissues from normal and glaucomatous donors (GSE27276) revealed antioxidant activity as one of the most markedly enriched molecular function [17]. Furthermore, mitochondrial dysfunction was evident in our mechanical stretch-induced glaucoma model [9], in agreement with clinical observations of mitochondrial gene variants in POAG patients [7] and mitochondrial abnormalities in glaucomatous TM cells [5]. Collectively, these results reinforce the concept that redox imbalance drives TM degeneration. In the present study, we extend this concept by showing that redox homeostasis is disrupted in both NRF2-deficient TM cells and NRF2 KO mice. NRF2 silencing led to superoxide accumulation, increased ROS production, redox disequilibrium, and elevated MDA levels (Fig. 2C to G). Conversely, NRF2 overexpression mitigated tBHP-induced oxidative damage (Fig. 2K to O), indicating its essential role in maintaining redox balance in TM cells. Importantly, NRF2 dysfunction is not merely an experimental observation but a feature of glaucoma pathology. NRF2 expression and activity are altered in glaucomatous TM cells [18] and in key ocular cell types in animal models of glaucoma, including RGCs and glia [19]. Consequently, therapeutic strategies aimed at reactivating NRF2 signaling have demonstrated efficacy in mitigating oxidative damage and providing neuroprotection in experimental glaucoma [20–22]. Together, these lines of evidence position the enhancement of NRF2 function in the TM as a compelling and mechanism-based therapeutic strategy for glaucoma.

The relevance of NRF2 and mitochondrial function under various diseases has been well-investigated. Studies show that novel NRF2 activators, such as chalcone derivatives, have been shown to mitigate mitochondrial dysfunction and tissue toxicity by binding to Keap1 and stabilizing NRF2 [23]. Furthermore, specific phytochemicals like isoliquiritigenin have demonstrated marked efficacy in attenuating nephrotoxicity, ototoxicity, and cerebral ischemia–reperfusion injury through activation of the Keap1–NRF2–ARE pathway, leading to reduced oxidative stress and improved mitochondrial function [24,25]. This pattern is highly consistent with the established role of NRF2 as a master regulator of inducible cytoprotection rather than a constitutive maintainer of core housekeeping functions. Our observation that NRF2 deficiency specifically impairs maximal respiratory capacity and spare respiratory capacity without affecting basal respiration and ATP linked respiration is consistent with its established role in regulating cellular bioenergetic flexibility. As demonstrated by Holmstrom et al. [10], NRF2 controls mitochondrial substrate availability, particularly glucose flux into the tricarboxylic acid (TCA) cycle, thereby determining the cell reserve capacity to cope with energetic demand and oxidative stress. This specialized role in stress adaptation further implies that the phenotypic consequences of NRF2 loss are highly context-dependent. Supporting this notion, studies have shown that global NRF2 deficiency can paradoxically improve glucose tolerance in a high-fat diet model through adipose tissue remodeling, yet simultaneously render the organism exquisitely sensitive to chemical hepatotoxicants [26]. This apparent paradox powerfully underscores that NRF2 is not a universal guardian of constitutive homeostasis but a master regulator of inducible defense, whose essential function becomes most apparent under conditions of specific metabolic or toxicological stress.

Our findings reveal that the mitochondrial dysfunction induced by NRF2 deficiency in TM cells is critically dependent on the down-regulation of NDUFS7, a core assembly and functional subunit of mitochondrial CI. This positions NDUFS7 as a pivotal mechanistic link between NRF2 signaling and cellular bioenergetics. NDUFS7 is ubiquitously expressed but shows varying levels across tissues, with high expression in metabolically active organs such as the heart and adrenal glands (https://www.ncbi.nlm.nih.gov/gene/374291). The expression and function of NDUFS7 are tightly regulated and have profound implications across diverse human diseases. In neurological disorders, NDUFS7 is indispensable for neuronal survival. Germline loss-of-function mutations cause Leigh syndrome by disrupting CI assembly, leading to severe bioenergetic failure in the basal ganglia and brainstem [27,28]. Its relevance extends to common neurodegenerative conditions, as decreased hippocampal NDUFS7 expression is identified as a biomarker associated with oxidative stress and mitochondrial dysfunction in Alzheimer’s disease [29]. Conversely, in oncology, NDUFS7 can play an opposite role. Elevated NDUFS7 expression supports the heightened metabolic demands of certain cancers, such as pancreatic ductal adenocarcinoma, where it has been validated as a therapeutic target and its pharmacologic inhibition selectively disrupts CI activity, inducing tumor cell death [30]. This dual role underscores that the cellular consequence of NDUFS7 dysregulation is context-dependent. Regulation of NDUFS7 occurs at multiple levels. The proper integration of NDUFS7 into CI at an early stage requires a hydroxylation modification that is carried out by the assembly helper protein NDUFAF5 [31]. In response to stress, NDUFS7 deficiency up-regulates the cystine transporter SLC7A11 to increase glutathione synthesis, revealing a compensatory antioxidant pathway that mitigates associated oxidative damage and ferroptosis [32]. While our data establish NRF2 as a crucial upstream regulator of NDUFS7 expression in TM cells, future studies should explore whether NRF2 also influences its posttranslational modification or assembly. In the context of glaucoma, the NRF2/NDUFS7 axis we identified in TM cells may represent a critical node linking oxidative stress, mitochondrial respiratory deficiency, and, potentially, IOP dysregulation. This aligns with the broader paradigm that fine-tuning the expression and function of core respiratory subunits like NDUFS7 is a central mechanism in both cellular health and disease pathogenesis.

Conventional glaucoma therapies predominantly target aqueous humor dynamics through IOP-lowering drugs or surgical interventions [33,34]. However, these treatments fail to address the chronic oxidative stress and mitochondrial dysfunction in TM cells. Given the central role of oxidative injury in TM degeneration and IOP dysregulation, therapeutic strategies aimed at restoring mitochondrial integrity and redox balance are urgently needed. Our findings identify the NRF2/NDUFS7 axis as a key modulator of TM mitochondrial health, suggesting that either enhancing NRF2 activity or directly augmenting NDUFS7 expression may offer a dual approach to alleviate oxidative stress and bioenergetic deficits. Such interventions could confer neuroprotective benefits beyond IOP control, preserving TM cell viability and function in glaucomatous eyes. To translate these findings into clinical practice, targeting the NRF2/NDUFS7 axis could be envisioned through several complementary strategies. Firstly, sulforaphane, a phytochemical derived from cruciferous vegetables, can activate NRF2 and is currently under clinical evaluation for neurodegenerative diseases such as Parkinson’s and Alzheimer’s [35,36]. Given the shared mechanisms of oxidative damage in neurodegeneration and glaucoma, repurposing NRF2 activators for glaucoma may accelerate translational progress. Preclinical studies should assess whether systemic or topical NRF2 activators can restore TM mitochondrial function, attenuate oxidative damage, and prevent chronic IOP elevation in glaucoma models. Second, a more targeted approach could involve gene therapy to enhance NDUFS7 expression specifically in the TM. Gene therapies leveraging adeno-associated virus (AAV) vectors have seen growing application in treating ocular disorders. A notable example is voretigene neparvovec (Luxturna), which in 2017 became the first Food and Drug Administration-approved AAV-based gene therapy for an inherited retinal disease, specifically Leber congenital amaurosis (https://www.fda.gov/media/110606/download). Delivering a functional NDUFS7 gene via AAV could directly rescue the CI assembly and bioenergetic defects caused by NRF2 deficiency, offering a potentially durable therapeutic effect. Finally, the NRF2/NDUFS7 axis may serve as a biomarker for patient stratification. Similar to the search for systemic biomarkers in glaucoma [37], measuring key components of this axis in accessible ocular tissues could identify patients with predominant TM mitochondrial dysfunction. This stratification would enable personalized therapeutic strategies targeting oxidative stress and bioenergetics, beyond conventional IOP lowering.

**Fig. 7.**
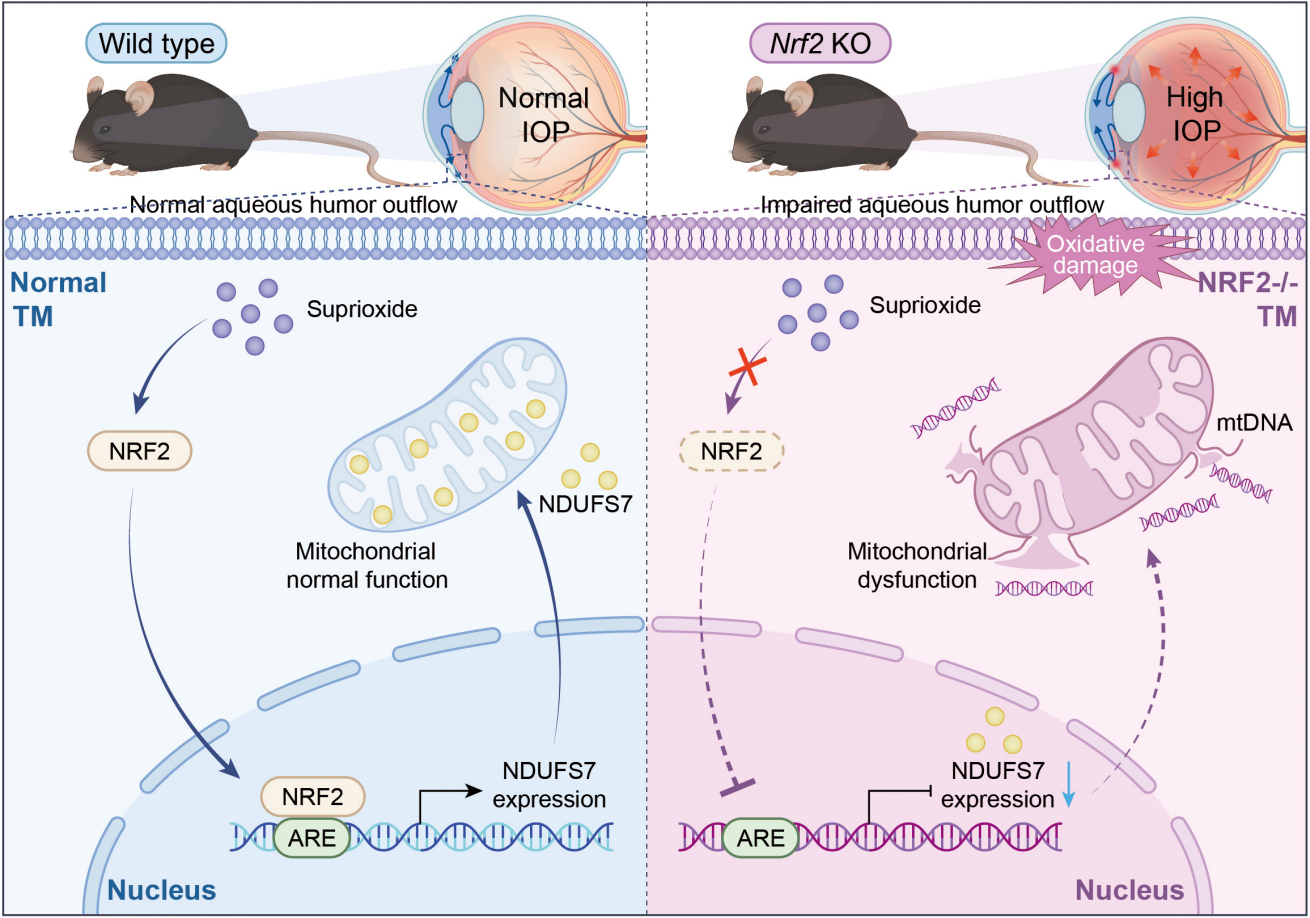
Schematic diagram of mechanism of NRF2 regulates NDUFS7 to influence mitochondrial homeostasis and TM function. In normal TM (left panel), in response to superoxide, NRF2 translocates to the nucleus, binds to the antioxidant response element (ARE), and promotes the expression of NDUFS7. NDUFS7 contributes to maintain normal mitochondrial function, thereby ensuring normal aqueous humor outflow and sustaining normal IOP. In NRF2-deficient TM (right panel), the activation of NRF2 in the TM is impaired, leading to decreased NDUFS7 expression, mitochondrial dysfunction, and oxidative damage. These abnormalities result in impaired aqueous humor outflow and elevated IOP.

In summary, our findings reveal that NRF2 safeguards TM cells by maintaining mitochondrial integrity through direct regulation of NDUFS7. Disruption of this NRF2/NDUFS7 axis results in oxidative injury, bioenergetic collapse, and TM dysfunction, ultimately contributing to glaucoma-associated ocular hypertension (Fig. 7). These insights deepen the mechanistic understanding of TM pathophysiology and identify NRF2 and NDUFS7 as promising therapeutic targets for preserving TM function in glaucoma.

## Materials and Methods

### Generation of *Nrf2* KO mice

The experimental procedures received approval and were monitored by the Institutional Animal Care and Use Committee of Capital Medical University, Beijing (AEEI-2023-310). All experimental procedures adhered to the NIH Guidelines for the Care and Use of Laboratory Animals.

CRISPR/Cas9-mediated genome editing was employed to generate the Nrf2 KO mouse model, disrupting the protein-coding reading frame of the *Nrf2* gene through direct deletion of exon 2 to exon 5 to produce a truncated fragment and subsequent loss of function. In brief, Cas9 mRNA and single-guide RNA (sgRNA) targeting critical exon regions essential for NRF2 function were synthesized via in vitro transcription. Fertilized C57BL/6J zygotes received co-microinjection of Cas9 mRNA and sgRNA, and the viable embryos were subsequently implanted into foster mothers that had been induced to a pseudo-pregnant state to generate F0 founder animals.

Potential founders were screened by PCR amplification of the targeted region followed by Sanger sequencing to confirm deletion. F0 mice harboring truncated fragment in the *Nrf2* gene were mated to WT C57BL/6J partners to test germline transmission. Heterozygous F1 offspring carrying validated mutations were identified through genotyping and sequencing. These F1 mice were interbred to obtain homozygous *Nrf2* KO mice for subsequent phenotypic and functional analyses.

### Human and nonhuman primate tissue

The corneoscleral ring tissue were obtained from the Eye Bank of Beijing Tongren Hospital. These tissues were surgical remnants from corneal transplantation procedures and were used for the present study. The use of these tissues was approved by the Ethics Committee of Beijing Tongren Hospital (Ethics No. TREC2025-KY138).

The rhesus monkey samples used in this study were acquired from Joinn Laboratories and handled in accordance with protocols approved by the Animal Ethics Committee (IACUC) of Joinn Laboratories (Suzhou) Co., Ltd. (IACUC Approval No. ACU21-2132).

### Evaluation of aqueous humor dynamics

Aqueous humor dynamics were evaluated using our previously established protocol [38]. Briefly, gadolinium contrast-enhanced magnetic resonance imaging (Gd-MRI) was employed to visualize aqueous humor flow. Animals were kept under 2% isoflurane anesthesia while respiratory rates were continuously monitored using a pneumatic sensor. After baseline image acquisition, gadolinium-DTPA (0.3 mmol/kg) was administered intraperitoneally, followed by sequential imaging at 10-min intervals over a 60-min period. Region-of-interest signal intensities were quantified in ImageJ and normalized to baseline values.

### IOP measurement

IOP determinations were carried out using the TonoLab tonometry apparatus. Mice were anesthetized with 5% isoflurane for 5 min, and IOP readings were obtained bilaterally with the handheld device. Each reported value represented the average of 3 consecutive measurements, with each measurement being the mean of 5 readings.

### Immunofluorescence staining

Paraformaldehyde-fixed, paraffin-embedded tissue specimens underwent xylene-based deparaffinization and graded ethanol rehydration. Epitope retrieval was carried out by immersing sections in sodium citrate buffer within a 95 °C water bath for 20 min. Following cooling to room temperature, sections were blocked for 60 min using 5% normal goat serum in phosphate-buffered saline (PBS) to mitigate nonspecific binding. Subsequently, an overnight incubation was conducted at 4 °C with primary antibodies, which were then detected using species-matched Alexa Fluor-conjugated secondary antibodies. After nuclear counterstaining with 4′,6-diamidino-2-phenylindole (DAPI), the samples were visualized, and fluorescence images were acquired with a Leica confocal microscope. The antibodies used included NRF2 (12721, CST), MDA (NBP2-59367, NOVUSBIO), NDUFS7 (PA5-106367, Thermo Fisher Scientific), α-SMA (67735-1-lg, Proteintech; 19245, CST), Alexa Fluor 488 (A21206, Thermo Fisher Scientific), and Alexa Fluor 546 (A10036, Thermo Fisher Scientific).

### TUNEL staining

Apoptotic cells were identified using a TUNEL assay kit (Vazyme Biotech, Cat# A112-01) following the manufacturer’s instructions with minor modifications. Paraformaldehyde-fixed, paraffin-embedded sections were deparaffinized in xylene and rehydrated through graded ethanol. After proteinase K digestion, sections were equilibrated in 100 μl of buffer for 30 min at room temperature, followed by enzymatic labeling with 50 μl of TdT reaction mix for 60 min at 37 °C in a humidified chamber. After 3 PBS washes (5 min each), nuclei were counterstained with DAPI to facilitate visualization. Fluorescence was captured on a Leica confocal microscope.

### Cell culture

Primary human TM cells (ScienCell, Carlsbad, CA, USA; Lots #5987 and #16873) were cultured in the supplier’s recommended medium (TMCM), containing 2% fetal bovine serum, 1% TM growth supplement, and 1% penicillin–streptomycin. All subsequent assays were conducted with cells at early passage (below passage 6).

### tBHP treatment

For oxidative stress induction, TM cells were treated with tBHP (Sigma-Aldrich, Cat# 458139) for 24 h. tBHP was diluted in complete culture medium to the desired working concentration.

### *NRF2* interference

TM cells were subjected to NRF2 knockdown using siRNA synthesized by Huzhou Hippo Biotechnology. TM cells were seeded at 2.0 × 10^5^ per well in 6-well plates and grown for 24 h prior to transfection to achieve 60% to 80% confluence. RNA–lipid complexes were prepared and applied to the medium, and cells were collected 48 h later to evaluate knockdown efficiency. The siRNA sequences were as follows: siCtrl: sense 5′-UUCUCCGAACGUGUCACGUdTdT-3′; antisense 5′-ACGUGACACGUUCGGAGAAdTdT-3′. siNRF2: sense 5′-GGUUGAGACUACCAUGGUUTT-3′; antisense 5′-AACCAUGGUAGUCUCAACCAG-3′.

### Quantitative real-time PCR

Total cellular RNA was extracted using TRIzol reagent (Invitrogen, Carlsbad, CA) following the manufacturer’s protocol. RNA purity and concentration were determined by absorbance at 260/280 nm using a PerkinElmer EnSpire multimode plate reader. First-strand cDNA synthesis was performed using 1 μg of total RNA and the Vazyme Reverse Transcription Kit (R323-01). Quantitative real-time PCR (qRT-PCR) was carried out in 20-μl volumes on an ABI 7500 system, employing Vazyme SYBR Green Master Mix (Q711-02) and gene-specific primers. The thermal cycling protocol began with a 30-s denaturation at 95 °C, followed by 40 cycles of 95 °C for 10 s and 60 °C for 30 s. For data analysis, the 2^−ΔΔCt^ method was used, with β-actin as the endogenous reference gene for normalization. Primer sequences are listed in Table S1.

### Western blotting

Protein expression was evaluated by immunoblotting. Cells were homogenized in ice-cold radioimmunoprecipitation assay buffer containing a protease-inhibitor cocktail. Following quantification with the Pierce Bicinchoninic Acid (BCA) kit (Thermo Fisher Scientific ), 30 μg of protein per sample was separated on 10% sodium dodecyl sulfate–polyacrylamide gel electrophoresis gels and electro-transferred to polyvinylidene fluoride (PVDF) membranes (Millipore). After blocking in 5% nonfat milk Tris-buffered saline containing 0.1% Tween-20 (TBST) for 1 h, membranes were incubated overnight at 4 °C with primary antibodies, followed by horseradish peroxidase (HRP)-conjugated secondary antibodies for 1 h at room temperature. Protein bands were visualized using a Bio-Rad ChemiDoc XRS imaging system. Antibodies used were listed as follows: NRF2 (12721, CST), NDUFS7 (PA5-106367, Thermo Fisher Scientific), GSR (SC-133245, Santa Cruz Biotechnology), SLC7A11 (ab307601, Abcam), HMOX1 (10701-1-AP, Proteintech), GCLM (14241-1-AP, Proteintech), NQO1 (11451-1-AP, Proteintech), GCLC (12601-1-AP, Proteintech), GPX4 (67763-1-Ig, Proteintech), GAPDH (SC-137179, Santa Cruz Biotechnology), and β-actin (SC-47778, Santa Cruz Biotechnology). HRP-conjugated anti-rabbit immunoglobulin G (IgG; 7074, CST) and anti-mouse IgG (ab205719, Abcam) served as secondary antibodies.

### DHE staining

ROS generation was assessed using DHE staining. DHE (Beyotime, S0064) was dissolved in dimethyl sulfoxide (DMSO) to prepare a 1 mM stock solution. TM cells were seeded in 6-well plates and grown to a confluency of 60% to 80% prior to subsequent procedures. Following treatment, cells were loaded with 5 μM DHE for 30 min at 37 °C, followed by 3 PBS washes to eliminate any residual dye. Fluorescence was visualized on a Zeiss microscope (excitation 535 nm). 

### BODIPY staining

BODIPY staining was employed to evaluate the cellular redox state. BODIPY (Thermo Fisher Scientific) was dissolved in DMSO to prepare a 10 mM stock solution. TM cells in 6-well plates were treated with 10 μM BODIPY for 30 min at 37 °C, washed three times with PBS, counterstained with Hoechst 33342 (Beyotime, C1022), and imaged on a Zeiss microscope (Ex 488/546 nm) to evaluate redox state.

### Measurement of MDA concentration

Lipid peroxidation was quantified by measuring MDA levels using an MDA Assay Kit according to the manufacturer’s protocol. Following treatment, cells were lysed in extraction buffer via ultrasonication. The resulting lysates were then vortexed and subjected to heat denaturation in a boiling water bath (≥95 °C) for 40 min, cooled promptly under running water, and clarified by centrifugation at 4,000 × *g* for 10 min, and a blank scan of the empty 96-well microplate was performed at 530 nm prior to measurement. Subsequently, 250 μl of each sample supernatant was loaded into the wells in triplicate. Absorbance was recorded using a microplate reader, and blank readings were subtracted from sample values to calculate final MDA concentrations.

### Measurement of intracellular GSH and GSSG levels

GSH and GSSG levels were measured using commercial assay kits purchased from Nanjing Jiancheng Bioengineering Institute (Nanjing, China) according to the manufacturer’s instructions. Briefly, cells were collected and washed twice with cold PBS, followed by lysis in the provided extraction buffer. Cell lysates were centrifuged to remove insoluble debris, and the resulting supernatants were used for subsequent analysis.

For GSH measurement, samples were incubated with the corresponding reaction reagents, and absorbance was measured using a microplate reader at the indicated wavelength. For GSSG determination, free GSH was selectively masked prior to the assay, allowing specific quantification of oxidized glutathione. GSSG concentrations were calculated based on standard curves generated according to the manufacturer’s protocol.

### Cell viability detection

Cell viability was detected using Cell Counting Kit-8 (CCK-8). TM cells (5 × 10^3^ per well) were plated in 96-well plates with 100 μl of complete medium and equilibrated overnight in a 5% CO₂ incubator at 37 °C. Thereafter, 10 μl of CCK-8 reagent was gently introduced into each well, ensuring that the cell monolayer remained undisturbed. Following 2 h of incubation under standard culture conditions, we measured the absorbance at 450 nm using a microplate reader.

### RNA sequencing

RNA quality was assessed with the RNA Nano 6000 Kit on an Agilent 2100 Bioanalyzer (Agilent Technologies, CA, USA). For library construction, 2 μg of total RNA per sample was applied with the NEBNext Ultra RNA Library Prep Kit for Illumina (New England Biolabs, USA) according to the manufacturer’s instructions. The resulting libraries underwent cluster amplification and sequencing on an Illumina HiSeq 4000 platform to yield 150-bp paired-end reads.

After quality filtering, clean reads were aligned to the reference genome, and only reads with exact matches or single-nucleotide mismatches were retained for downstream gene annotation.

The screening for DEGs was conducted in R with the DESeq package (v1.10.1). We applied the Benjamini–Hochberg procedure to control the false discovery rate (FDR) and defined statistical significance for gene expression changes as an adjusted *P* value of less than 0.05.

GSEA was further applied to identify biologically relevant pathways. Genes were ranked according to their log_2_ fold change, and enrichment was evaluated using the MSigDB gene sets. Normalized enrichment scores were computed, and pathways with an FDR of <0.25 were regarded as markedly enriched.

### Mitochondrial membrane potential assessment by TMRE staining

Following staining with 100 nM TMRE (Abcam, ab113852) in culture medium (20 min, 37 °C), cells were rinsed twice with PBS to eliminate excess probe. Confocal microscopy was then immediately employed to capture the fluorescence signals, thereby minimizing any potential signal decay.

### Mitochondrial stress test

Mitochondrial respiration was evaluated with the Seahorse XF Cell Mito Stress Test Kit. We plated TM cells in XF24 microplates at 4 × 10^4^ per well and left them to adhere overnight. Before measurement, the medium was replaced with nonbuffered XF assay medium supplemented with appropriate energy substrates. After a 60-min incubation for pH equilibration in a non-CO₂ environment, real-time OCRs were recorded using the Seahorse XFe24 Extracellular Flux Analyzer. Sequential injections of mitochondrial modulators were automatically administered: oligomycin (1.5 μM, ATP synthase inhibitor), FCCP (2 μM, proton ionophore), and rotenone/antimycin A (0.5 μM, ETC inhibitors). Key bioenergetic parameters—including basal respiration, ATP production, proton leak, spare respiratory capacity, and maximal respiration—were determined following subtraction of non-mitochondrial respiration and normalized to cell number.

### Transmission electron microscopy

Following a 2-h primary fixation at room temperature with 2.5% glutaraldehyde, enucleated eyes were dissected. One quadrant of the anterior segment was reserved at 4 °C, rinsed thrice with PBS and subsequently postfixed in 1% osmium tetroxide for 2 h. A graded ethanol series (50%, 70%, 90%, and 100%) was used for dehydration prior to embedding in epoxy resin for ultrastructural examination.

### ChIP-seq assay

ChIP was performed using the BeyoChIP Enzymatic ChIP Assay Kit (Beyotime, P2083S) following the manufacturer’s protocol. Briefly, 1 × 10^7^ human trabecular meshwork (HTM) cells were fixed and resuspended in ChIP buffer containing 1× Protease Inhibitor Cocktail (CST). Chromatin was enzymatically sheared using micrococcal nuclease to generate DNA fragments between 150 and 900 bp. Immunoprecipitation was conducted overnight at 4 °C using 10 μl of pre-cleared protein A/G agarose beads with anti-NRF2 (Abcam, ab62352) or anti-IgG (Abcam, ab172730) antibodies. The recovered DNA fragments were sequenced on the NovaSeq platform, and NRF2-binding sites were visualized and annotated using IGV software.

### ChIP qPCR assay

Following ChIP, purified DNA was subjected to qPCR to verify NRF2 binding at the NDUFS7 promoter. The primer sequences were as follows: *NDUFS7* sense: 5′-GAGGCTGAGGCAGGAGAATC-3′. *NDUFS7* antisense: 5′-GAGATGGAGTCTTGCTCTGTCA-3′.

### Luciferase reporter assay

To assess *NDUFS7* promoter activity, 293T cells overexpressing NRF2 were cotransfected with recombinant plasmids harboring the NDUFS7 promoter-driven firefly luciferase construct and the pRL-null vector expressing Renilla luciferase as an internal control. Luminescence was detected with a FlexStation 3 microplate reader, and firefly signals were adjusted to Renilla activity to correct for transfection efficiency.

### Statistical analysis

All quantitative results are represented as mean ± SD from triplicate experiments. GraphPad Prism 11.0 was employed for statistical analysis. Intergroup differences were assessed using a 2-tailed Student *t* test for 2 groups, or one-way analysis of variance with Tukey’s post hoc test for multiple groups, considering *P* < 0.05 as statistically significant.

## Data Availability

All data are available in the main text or the Supplementary Materials.
