# Supplementary material for: NRF2 Deficiency Disrupts Mitochondrial Homeostasis via NDUFS7 in Trabecular Meshwork
Source: Research (Wash D C). 2026 Mar 20;9:1203. doi: 10.34133/research.1203 (PMC13003157; doi:10.34133/research.1203)
Supplement: Supplementary 1 — Figs. S1 to S4 Table S1 [file research.1203.f1.zip › Supplementary materials-new.docx]

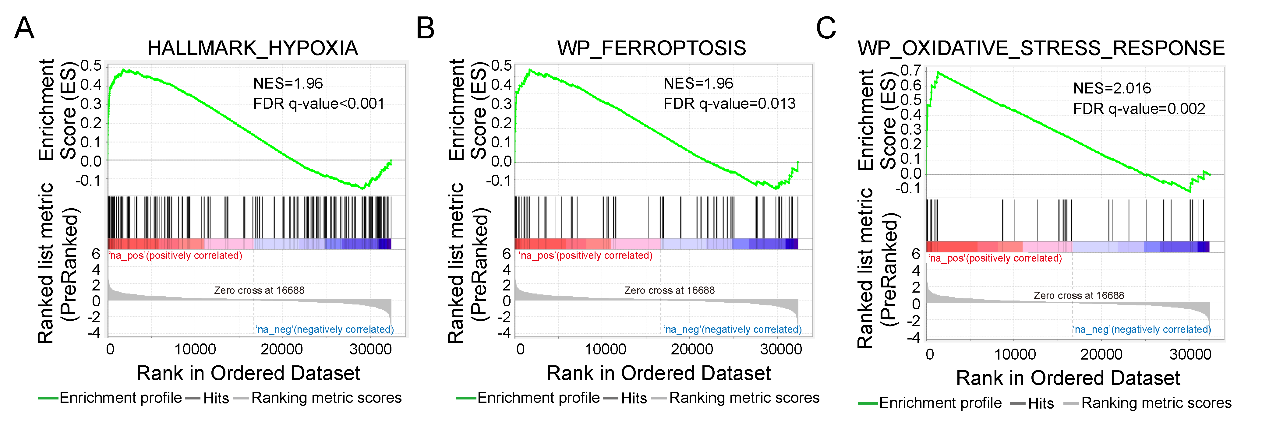


**Supplementary Figure 1. Gene set enrichment analysis (GSEA) indicates enrichment of oxidative injury related pathways.** (A) Enrichment of the hypoxia gene set. (B) Enrichment of the ferroptosis gene set. (C) Enrichment of the oxidative stress response gene set.


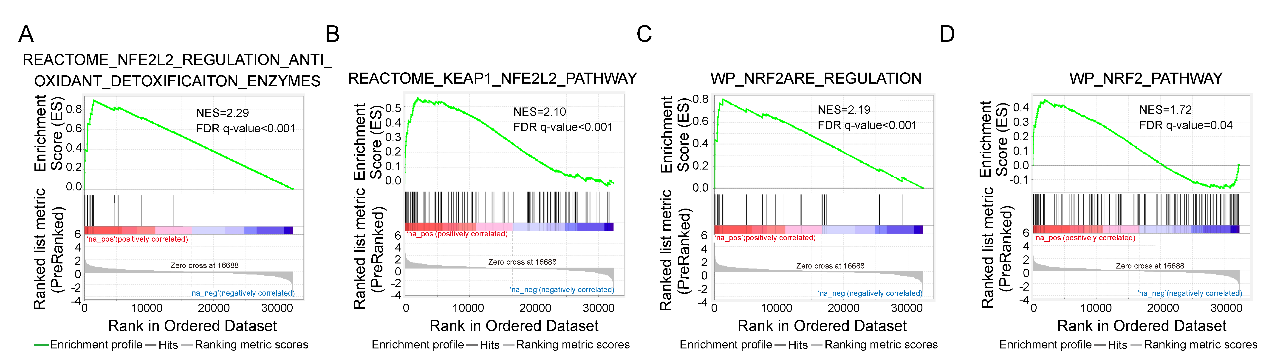


**Supplementary Figure 2. Gene set enrichment analysis (GSEA) reveals activation of the NRF2 signaling pathway.** (A) Enrichment of the NFE2L2 regulation of antioxidant detoxification enzymes gene set. (B) Enrichment of the KEAP1/NFE2L2 pathway gene set. (C) Enrichment of the NRF2 ARE regulation gene set. (D) Enrichment of the NRF2 pathway gene set.


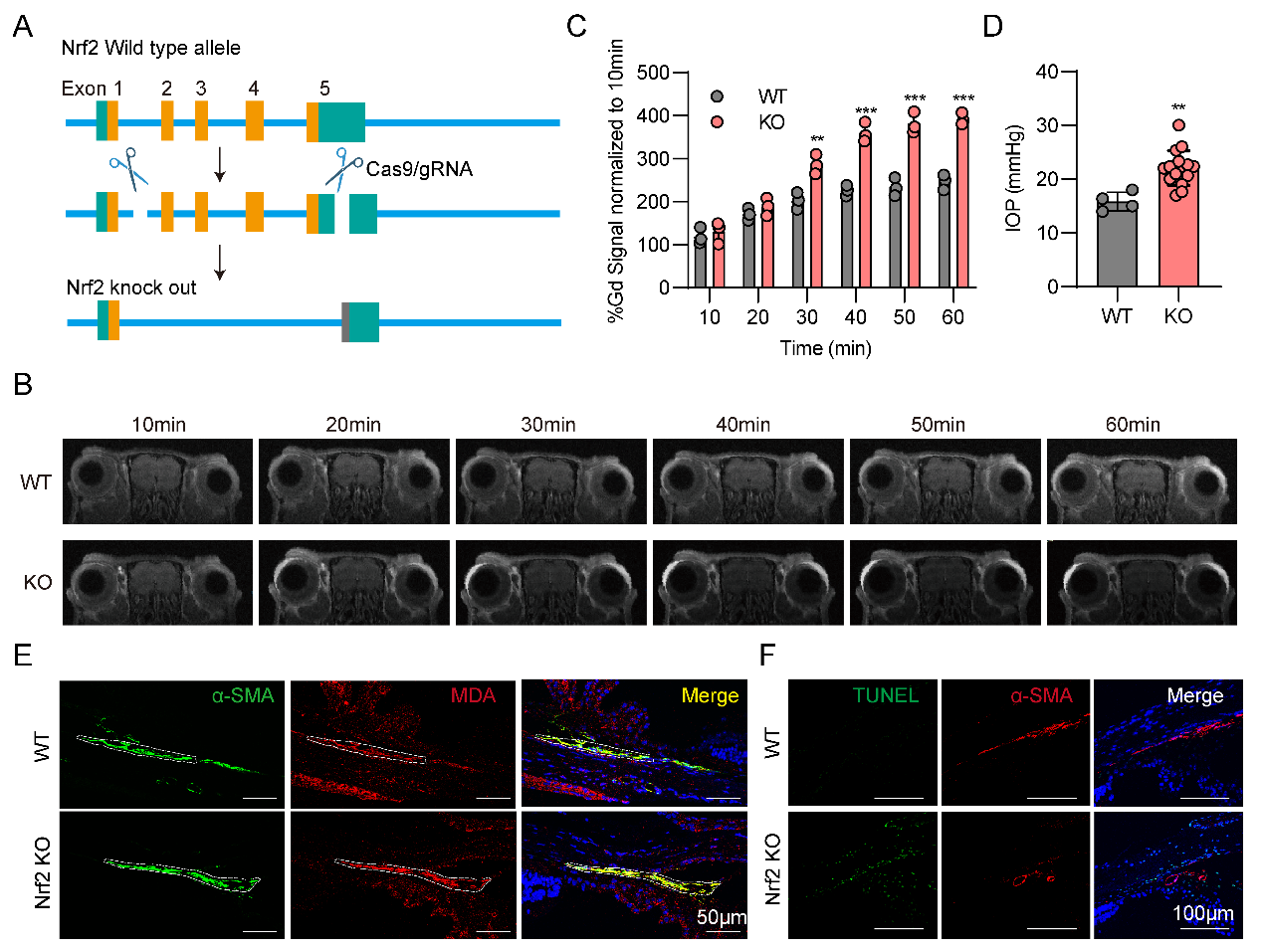


**Supplementary Figure 3. Loss of NRF2 induces TM injury.** (A) Schematic diagram of NRF2 knockout mice. (B) Gd-MRI monitoring of aqueous humor dynamics in WT and NRF2 KO mice. (C) Quantification of Gd-MRI signal intensity over time as shown in (B). ***P*<0.01, ****P*<0.001. (D) Intraocular pressure (IOP) measurements in WT (n=4) and 6-month-old NRF2 KO mice (n=14). ***P*<0.01. (E) Immunofluorescence staining of the TM marker α-SMA (green) and MDA (red) in the anterior chamber angle tissue in WT and NRF2 KO mice. (F) TUNEL staining images in the anterior chamber angle tissue in WT and NRF2 KO mice.


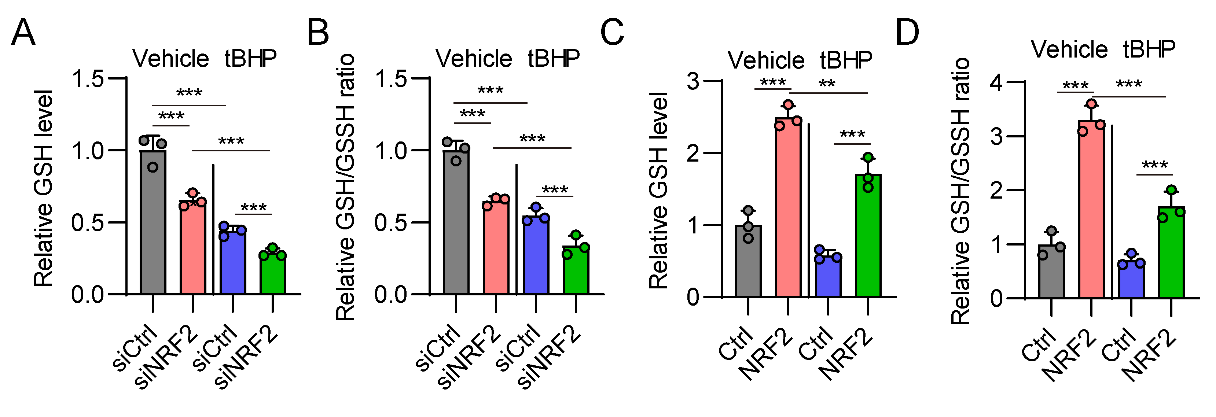


**Supplementary Figure 4. NRF2 regulates cellular glutathione redox homeostasis under basal and oxidative stress conditions.** (A) Relative intracellular GSH levels in TM cells under NRF2 knockdown with or without tBHP exposure. ***P<0.001. (B) Relative GSH/GSSG ratio in TM cells under NRF2 knockdown with or without tBHP exposure. ****P*<0.001. (C) Relative intracellular GSH levels in TM cells under NRF2 overexpression with or without tBHP exposure. ***P*<0.01, ***P<0.001. (D) Relative GSH/GSSG ratio in TM cells under NRF2 overexpression with or without tBHP exposure. ***P<0.001.
