# Supplementary material for: NRF2 Deficiency Disrupts Mitochondrial Homeostasis via NDUFS7 in Trabecular Meshwork
Source: Research (Wash D C). 2026 Mar 20;9:1203. doi: 10.34133/research.1203 (PMC13003157; doi:10.34133/research.1203)
Supplement: Supplementary 1 — Figs. S1 to S4 Table S1 [file research.1203.f1.zip › table 1.docx]

**Table.1**

| qPCR Primer Sequence | |  |  |
| --- | --- | --- | --- |
| Gene | Forward (5’-3’) | | Reverse (5’-3’) |
| Mouse *β-actin* | GGCTGTATTCCCCTCCATCG | | CCAGTTGGTAACAATGCCATGT |
| Mouse *Ndufs7* | GCTGGCACGCTTACCAACAAGA | | AGCCACGAACAACCGAGTAGGA |
| Mouse *Tuft1* | AAGTGGCTGGGTTTCGGGAGA | | TTCCGTGGCTGACCTGCTTCT |
| Mouse *Lamb3* | CCGCTTGCTGATGGAGGAAGAT | | TGCCTCGCTGACCTGTTGGA |
| Mouse *Uhrf2* | GGTGAGCGAAGCAGGTGTTCAT | | AGTGTCTGGTCAGCCGATGGAG |
| Mouse *Nqo1* | GGTAGCGGCTCCATGTACTCTC | | TCCTCCCAGACGGTTTCCAGAC |
| Mouse *Cd274* | AGCCTCAGCACAGCAACTTCAG | | CTTGTAGTCCGCACCACCGTAG |
| Mouse *Gpx4* | GCCCGATATGCTGAGTGTGGTT | | GCACGGCAGGTCCTTCTCTATC |
| Mouse *Plin2* | GCGAAGGATGTGGTGACGACTA | | GCTCCGACTTGGTGATGGCATT |
| Mouse *Adhfe1* | CAGTGCCCAACTCATTCTCAT | | ACTTCCTTCGTAACCCCTGCT |
| Mouse *Icam1* | GTGATGCTCAGGTATCCATCCA | | CACAGTTCTCAAAGCACAGCG |
| Human *β-actin* | CATGTACGTTGCTATCCAGGC | | CTCCTTAATGTCACGCACGAT |
| Human *NDUFS7* | CCGGCACACTCACCAACAAGAT | | TGTAGATGTCCACGGGCACGAT |
| Human *TUFT1* | CGGAGCAACAATGCTGACTG | | ATTTGCCGGACTTTCCGCT |
| Human *LAMB3* | GACTTCGGTAAGACCTGGCG | | CTCTCAAGTTTGTGATCTCCCC |
| Human *UHRF2* | GCCTTGGTGCTTGGTTTGAAG | | AGACGTAGAGGGTACACTGTC |
| Human *NQO1* | GAAGAGCACTGATCGTACTGGC | | GGATACTGAAAGTTCGCAGGG |
| Human *CD274* | AATTCGCTTGTAGTCGGCACC | | AATTCGCTTGTAGTCGGCACC |
| Human *GPX4* | GAGGCAAGACCGAAGTAAACTAC | | CCGAACTGGTTACACGGGAA |
| Human *PLIN2* | ATGGCATCCGTTGCAGTTGAT | | GGACATGAGGTCATACGTGGAG |
| Human *ADHFE1* | TGGACTTTCACCTTCTGGGAA | | GGAGAGGTTCTTGTCTGTCATCA |
| Human *ICAM1* | ATGCCCAGACATCTGTGTCC | | GGGGTCTCTATGCCCAACAA |
| Human *NRF2* | TTCCCGGTCACATCGAGAG | | TCCTGTTGCATACCGTCTAAATC |
| Human *GSR* | TTCCAGAATACCAACGTCAAAGG | | GTTTTCGGCCAGCAGCTATTG |
| Human *SLC7A11* | TCTCCAAAGGAGGTTACCTGC | | AGACTCCCCTCAGTAAAGTGAC |
| Human *HMOX1* | ACATTGCCAGTGCCACCAAGTT | | TGTTGAGCAGGAACGCAGTCTT |
| Human *GCLM* | CATTTACAGCCTTACTGGGAGG | | ATGCAGTCAAATCTGGTGGCA |
| Human *GCLC* | GGAGGAAACCAAGCGCCAT | | CTTGACGGCGTGGTAGATGT |
